# Breast Cancer Incidence Among US Women Aged 20 to 49 Years by Race, Stage, and Hormone Receptor Status

**DOI:** 10.1001/jamanetworkopen.2023.53331

**Published:** 2024-01-26

**Authors:** Shuai Xu, Sara Murtagh, Yunan Han, Fei Wan, Adetunji T. Toriola

**Affiliations:** 1 Department of Surgery, Division of Public Health Sciences, and Siteman Cancer Center, Washington University School of Medicine, St Louis, Missouri; 2 School of Medicine, University College Dublin, Belfield, Dublin, Ireland

## Abstract

**Importance:**

Breast cancer in young women has a less favorable prognosis compared with older women. Yet, comprehensive data on recent trends and how period and cohort effects may affect these trends among young women are not well-known.

**Objective:**

To evaluate breast cancer incidence among young women in the US over a 20-year period by race and ethnicity, hormone receptor status (estrogen receptor [ER] and progesterone receptor [PR]), tumor stage, and age at diagnosis, as well as how period and cohort effects may affect these trends.

**Design, Setting, and Participants:**

This cross-sectional study used data from Surveillance, Epidemiology, and End Results 17 registries (2000-2019). Women aged 20 to 49 years with a primary invasive breast cancer were included. Data were analyzed between February and June 2023.

**Main Outcomes and Measures:**

Age-standardized incidence rates (ASIR), incidence rate ratios (IRR), and average annual percent changes (AAPC) stratified by race and ethnicity, hormone receptor status, tumor stage, and age at diagnosis.

**Results:**

Out of 217 815 eligible women (1485 American Indian or Alaska Native [0.7%], 25 210 Asian or Pacific Islander [11.6%], 27 112 non-Hispanic Black [12.4%], 37 048 Hispanic [17.0%], 126 960 non-Hispanic White [58.3%]), the majority were diagnosed with an ER+/PR+ tumor (134 024 [61.5%]) and were diagnosed with a stage I tumor (81 793 [37.6%]). Overall, invasive breast cancer incidence increased (AAPC, 0.79; 95% CI, 0.42 to 1.15), with increasing trends across almost all racial and ethnic groups. ASIR increased for ER+/PR+ (AAPC, 2.72; 95% CI, 2.34 to 3.12) and ER+/PR− tumors (AAPC, 1.43; 95% CI, 1.00 to 1.87), and decreased for ER−/PR+ (AAPC, −3.25; 95% CI, −4.41 to −2.07) and ER−/PR− tumors (AAPC, −0.55; 95% CI, −1.68 to 0.60). For women aged 20 to 29 and 30 to 39 years, ASIRs were highest among non-Hispanic Black women (age 20-29 years: IRR, 1.53; 95% CI, 1.43 to 1.65; age 30-39 years: IRR, 1.15; 95% CI, 1.12 to 1.18). For women aged 40 to 49 years, ASIR was lower for non-Hispanic Black women (IRR, 0.96; 95% CI, 0.94 to 0.97) compared with non-Hispanic White women. Incidence rates increased for stages I and IV tumors but decreased for stage II and III tumors. Age-period-cohort analysis demonstrated both cohort and period effects on breast cancer incidence (*P* < .001).

**Conclusions and Relevance:**

In this population-based cross-sectional analysis, an increase in breast cancer incidence rates among young US women and age-related crossover between non-Hispanic White and Black women were observed. Prevention efforts in young women need to adopt a targeted approach to address racial disparities in incidence rates observed at different age phases.

## Key Points

**Question:**

What are the long-term trends in breast cancer incidence among women aged 20 to 49 years?

**Findings:**

In this population-based, cross-sectional study using data from Surveillance, Epidemiology, and End Results, age-standardized, age-cohort–adjusted, and age-period–adjusted breast cancer incidence rates increased over the past 20 years among different races in different age groups. Incidence rates for estrogen receptor (ER)-positive, stage I, and stage IV tumors increased, while rates decreased for ER-negative, stage II, and stage III tumors.

**Meaning:**

These results suggest that understanding factors driving differential trends in incidence rates for different age groups by race and ER-positive status should provide insights into breast cancer prevention in young women.

## Teaser

This cross-sectional study examines 20-year trends in breast cancer incidence rates by age, race and ethnicity, cancer stage, and hormone receptor status among US women aged 20 to 49 years.

## Introduction

Breast cancer is the most commonly diagnosed cancer and the leading cause of cancer death among women aged 20 to 49 years in the US.^1^ Young women are more likely to develop breast cancer with more aggressive biological features compared with older women, including larger tumor size, advanced tumor stage, negative hormone receptors status (estrogen receptor [ER] and progesterone receptor [PR]), and overexpression of the human epidermal growth factor receptor 2 (ERBB2; formerly HER2),^2,3,4^ all contributing to the poorer prognosis among young female patients with breast cancer. Additionally, except for high-risk women, breast cancer screening programs are not available for women aged under 40 years.

Recent studies found a rising trend in breast cancer incidence among young women,^4,5,6^ but few studies comprehensively described trend patterns by hormone receptors, stages, and races. Although previous studies used age-adjusted rates as a means for adjustment for age, there is still limited data on the differences caused by the unique social and ecological environment among populations born in the same year (cohort effects) and the social and environmental context that modifies risks for all young women at a particular calendar time (period effects) in the US. Furthermore, studies have shown that young non-Hispanic Black women are more likely to be diagnosed with breast cancer than non-Hispanic White women,^4^ especially biologically aggressive subtypes (triple-negative or ER-negative tumors),^7,8^ but most of these studies were published before 2020. Understanding these time effects and differences may inform the potential causes of breast cancer among young women for future research as well as offer insight into opportunities for prevention in this population.

To the best of our knowledge, there is limited data on breast cancer incidence trends across decades and the simultaneous effects of age, period, and cohort among young US women aged 20 to 49 years. We used data from the Surveillance, Epidemiology, and End Results (SEER) Program to calculate breast cancer incidence trends among this target population over the most recent 20 years and stratified our analyses by race and ethnicity, hormone receptor status, tumor stage, and age at diagnosis. Furthermore, we assessed how birth cohort and calendar period affected breast cancer incidence among these women.

## Methods

### Data Source

We analyzed data from the SEER 17 Registries covering 2000 to 2019 years for primary analysis. This database was subleased in November 2021 and covers nearly 27% of the US population. Due to the use of deidentified data, this study was determined as exempt from review and informed consent requirements by the institutional review board of Washington University School of Medicine in St Louis. We followed the Strengthening the Reporting of Observational Studies in Epidemiology (STROBE) guideline for cross-sectional studies.

### Study Population

We included women aged between 20 and 49 years who had been diagnosed with primary invasive breast cancer according to the *International Classification of Diseases for Oncology, Third Edition* (*ICD-O-3*). Inclusion criteria screened for patients who were female; between ages 20 and 49 years; diagnosed with primary invasive breast cancer between 2000 and 2019; with stage I to IV, unstaged, or unknown disease; and with available information on race. From 302 216 potentially eligible female participants, we excluded 29 767 women with an unknown or a prior cancer history, 53 251 women with stage 0 breast cancer, and 1383 women with unknown race. The final analytic set included 217 815 women.

Age at diagnosis was formatted as 5-year age groups (20-24, 25-29, 20-34, 35-39, 40-44, and 45-49 years). Age-standardized incidence rates (ASIR) were calculated using these 6 age subgroups. Age at diagnosis was categorized into 3 ten-year age subgroups (20-29, 30-39, and 40-49 years) for analyses of the age differences in breast cancer incidence rates and trends. We defined hormone receptor status (HR) as the joint expression of ER and PR. If either ER or PR status was borderline or unknown, HR was recorded as unknown. Thus, ER and PR were grouped into 4 HR categories (ER+/PR+; ER+/PR−; ER−/PR+; ER−/PR−; and unknown). We used SEER-derived race and ethnicity subgroups: Hispanic, non-Hispanic American Indian or Alaska Native, non-Hispanic Asian or Pacific Islander, non-Hispanic Black, and non-Hispanic White.

We applied the American Joint Committee on Cancer (AJCC) staging algorithms to determine the stage. We used Breast-Adjusted AJCC 6th Stage for data from 2000 to 2015, Derived SEER Combined Stage Group for 2016 and 2017, and Derived Extent of Disease for 2018 and after. Tumor stage was categorized into 5 subgroups: stages I through IV and unknown or unstaged. ASIR of breast cancer was the primary outcome. We calculated ASIR (expressed as cases per 100 000 women), incidence rate ratios (IRR) and their 95% CIs based on 2000 US standard population using SEER*Stat version 8.4.2 (National Cancer Institute). IRRs were estimated using Tiwari modification.^9^

### Statistical Analysis

To explore the potential heterogeneity in incidence rates, we performed subgroup analyses and computed ASIRs by race and ethnicity, HR, and stage. To compare the incidence rates between groups, we then computed IRRs with non-Hispanic White, ER+/PR+, and stage I as reference groups. To further explore the effects of age at diagnosis and HR on the potential heterogeneity in the ASIRs of different race and ethnicities, we computed ASIR and IRR of race and ethnicity categories within each age group and each age and HR category. The relevant analyses were performed using SEER*Stat version 8.4.2.

To investigate the potential nonlinear trends in ASIRs over the period 2000 to 2019, we fitted Joinpoint regressions to model the natural log transformed annual ASIRs. Joinpoint regression starts with a straight line (0 joinpoint), and tests whether more statistically significant joinpoints need to be added to the model based on the Monte Carlo Permutation method.^10^ We allowed a maximum of 3 joinpoints per model. Using the fitted Joinpoint models, we computed the average annual percent change (AAPC) to measure the average trend over 2000 to 2019. AAPC is computed as a weighted average of the annual percent changes (APC) for each identified segment from the joinpoint model, with the weights equal to the length of the segment intervals over which APCs are computed. AAPC is still valid even when the joinpoint model reveals the changes in trends in ASIRs over 2000 to 2019.^11^

To evaluate the heterogeneity in trends, we repeated Joinpoint analysis by race and ethnicity, stage, and HR. Joinpoint analyses were implemented in NCI Joinpoint regression software version 5.0.2 (National Cancer Institute). AAPC and APC confidence intervals were estimated by the parametric method.

We used the National Cancer Institute’s Age-Period-Cohort web tool to build age-period-cohort models to account for the age, calendar period, and birth cohort effects on the trends in breast cancer incidence.^12^ Wald χ^2^ tests were performed to determine whether period and cohort impacted the trends. We formatted the input data into 4 five-year time periods (2000-2004, 2005-2009, 2010-2014, 2015-2019) and 6 five-year age groups (20-24, 25-29, 30-34, 35-39, 40-44, 45-49), spanning 9 birth cohorts (from 1955 to 1995 in 5-year intervals). The cohort and period effects are presented as IRRs using 1955 cohort and period 2000-2004 as the reference groups, respectively. We performed analyses stratified by race and ethnicity, HR, and stage.

We also performed sensitive analysis in the SEER 12 data set that covered years 1995 to 2019 and in the SEER 22 database covering years 2000 to 2019. SEER 12 and 22 databases cover approximately 12% and 48% of the US population. There were no major differences in breast cancer incidence patterns between SEER 12 or SEER 22 and SEER 17 (data not shown). All statistical tests were 2-sided, and *P* values <.05 were considered statistically significant.

## Results

### Characteristics of the Study Population

In total, 217 815 women aged 20 to 49 years diagnosed with primary invasive breast cancer from 2000 to 2019 were included in these analyses (1485 American Indian or Alaska Native [0.7%], 25 210 Asian or Pacific Islander [11.6%], 27 112 non-Hispanic Black [12.4%], 37 048 Hispanic [17.0%], 126 960 non-Hispanic White [58.3%]) (Table 1). The majority were non-Hispanic White (126 960 [58.3%]), diagnosed with an ER+/PR+ tumor (134 024 [61.5%]), and with a stage I tumor (81 793 [37.6%]). A small proportion of participants had missing data on HR (18 077 [8.3%]) and tumor stage (12 832 [5.9%]).

**Table 1.** Trends in Breast Cancer Incidence Among Women Aged 20-49 Years by Race and Ethnicity, Hormone Receptor Status, and Cancer Stage, 2000-2019

Characteristics	Total, No. (%)	Age-adjusted incidence rates, No. per 100 000	Incidence rate ratios (95% CI)	Average annual change, % (95% CI)^a^
Overall	217 815 (100)	65.8	NA	0.79 (0.42 to 1.15)
Race and ethnicity				
Hispanic	37 048 (17.0)	52.8	0.76 (0.75 to 0.77)	0.80 (0.39 to 1.21)
Non-Hispanic American Indian or Alaska Native	1485 (0.7)	52.6	0.75 (0.72 to 0.79)	1.66 (0.59 to 2.75)
Non-Hispanic Asian or Pacific Islander	25 210 (11.6)	67.0	0.96 (0.95 to 0.97)	1.52 (0.93 to 2.11)
Non-Hispanic Black	27 112 (12.4)	70.7	1.01 (1.00 to 1.03)	0.10 (−0.48 to 0.69)
Non-Hispanic White	126 960 (58.3)	69.7	1 [Reference]	0.85 (0.55 to 1.15)
Hormone receptor status				
ER+/PR+	134 024 (61.5)	40.5	1 [Reference]	2.72 (2.34 to 3.12)
ER+/PR−	17 123 (7.9)	5.2	0.13 (0.13 to 0.13)	1.43 (1.00 to 1.87)
ER−/PR+	3506 (1.6)	1.1	0.03 (0.03 to 0.03)	−3.25 (−4.41 to −2.07)
ER−/PR−	45 085 (20.7)	13.7	0.34 (0.33 to 0.34)	−0.55 (−1.68 to 0.60)
Unknown^b^	18 077 (8.3)	5.5	0.13 (0.13 to 0.14)	−6.31 (−9.82 to −2.66)
AJCC stage^c^				
Stage I	81 793 (37.6)	24.7	1 [Reference]	3.45 (2.32 to 4.60)
Stage II	81 255 (37.3)	24.6	1.00 (0.99 to 1.01)	−3.42 (−5.18 to −1.63)
Stage III	32 424 (14.9)	9.8	0.40 (0.39 to 0.40)	−3.06 (−4.24 to −1.86)
Stage IV	9511 (4.4)	2.9	0.12 (0.11 to 0.12)	3.39 (2.92 to 3.87)
Unknown or unstaged	12 832 (5.9)	3.9	0.16 (0.15 to 0.16)	5.78 (1.11 to 10.66)

Abbreviations: AJCC, American Joint Committee on Cancer; ER, estrogen receptor; NA, not applicable; PR, progesterone receptor.

^a^ Average annual percent change was calculated in weighted sum of annual percent change sections.

^b^ Either estrogen or progesterone receptor status are unknown, borderline, or not available.

^c^ Breast-Adjusted AJCC stage was used from 2000 to 2015, derived SEER CMB STG GRP (combined stage group) was used for 2016 to 2017, and derived EOD (extent of disease) 2018 stage group was used for 2018 and 2019.

### Overall Breast Cancer Incidence Rates and Trends

**Figure 1.**
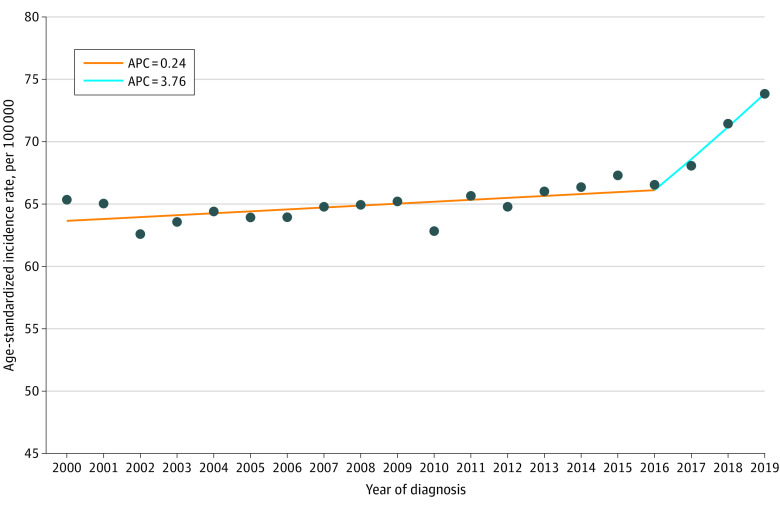
Joinpoint Analysis of Overall Incidence of Primary Invasive Breast Cancer Among US Women Aged 20 to 49 Years, 2000-2019 APC indicates annual percent changes.

Differences in ASIRs, IRRs, and AAPCs by race and ethnicity, stage, and HR are shown in Table 1. Overall, breast cancer incidence rates increased by 0.79% (95% CI, 0.42 to 1.15) annually over the study period. Incidence increased gradually (APC, 0.24; 95% CI, 0.05 to 0.42) between 2000 and 2016 and then dramatically from 2016 onwards (APC, 3.76; 95% CI, 1.39 to 6.19) (Figure 1). ASIRs were highest among non-Hispanic Black (70.7 patients per 100 000) and non-Hispanic White (69.7 patients per 100 000) women and lowest among non-Hispanic American Indian or Alaska Native (52.6 patients per 100 000) and Hispanic (52.8 patients per 100 000) women. ASIR was highest for ER+/PR+ tumors (40.5 patients per 100 000) and lowest for ER−/PR+ tumors (1.1 patients per 100 000). ASIR increased for ER+/PR+ (AAPC, 2.72; 95% CI, 2.34 to 3.12) and ER+/PR− tumors (AAPC, 1.43; 95% CI, 1.00 to 1.87), and decreased for ER−/PR+ (AAPC, −3.25; 95% CI, −4.41 to −2.07). ASIR increased for stages I and IV tumors but decreased for stages II and III tumors. The joinpoints for race and ethnicity, HR, and stage are shown in eFigures 1, 2, and 3 in Supplement 1, respectively.

### Differences in Breast Cancer Incidence Rates by Age Group and Race

Among women aged 20 to 29 years, ASIR was highest among non-Hispanic Black women (IRR, 1.53; 95% CI, 1.43 to 1.65) and lowest among Hispanic women (IRR, 0.87; 95% CI, 0.81 to 0.93) (Table 2). Significant increases in ASIR during the study period occurred among non-Hispanic White (AAPC, 1.64; 95% CI, 1.17 to 2.11), non-Hispanic Asian or Pacific Islanders (AAPC, 1.65; 95% CI, 0.22 to 3.09) and Hispanic women (AAPC, 1.31; 95% CI, 0.46 to 2.18). Among women aged 30 to 39 years, ASIR was highest among non-Hispanic Black women (IRR, 1.15; 95% CI, 1.12 to 1.18) and lowest among Hispanic women (IRR, 0.76; 95% CI, 0.74 to 0.78). Significant increases in ASIR occurred among Hispanic (AAPC, 1.06; 95% CI, 0.55 to 1.58), non-Hispanic Black (AAPC, 0.54; 95% CI, 0.14 to 0.93), and non-Hispanic White women (AAPC, 0.56; 95% CI, 0.34 to 0.77). Among women aged 40 to 49 years, ASIR was lower in non-Hispanic Black women (IRR, 0.96; 95% CI, 0.94 to 0.97) compared with non-Hispanic White and lowest among non-Hispanic American Indian and Alaska Native women (IRR, 0.72; 95% CI, 0.68 to 0.76).

**Table 2.** Trends in Breast Cancer Incidence Among Women Aged 20-49 Years by Race, Ethnicity, and Age, 2000-2019

Characteristics	Total, No. (%)	Age-adjusted incidence rates, No. per 100 000	Incidence rate ratios (95% CI)	Average annual change, % (95% CI)^a^
**Age 20-29 y**				
Hispanic	1298 (22.8)	4.2	0.87 (0.81 to 0.93)	1.31 (0.46 to 2.18)
Non-Hispanic American Indian or Alaska Native	48 (0.8)	4.5	0.93 (0.68 to 1.23)	NA^b^
Non-Hispanic Asian or Pacific Islander	578 (10.2)	4.4	0.90 (0.82 to 0.98)	1.65 (0.22 to 3.09)
Non-Hispanic Black	1055 (18.6)	7.5	1.53 (1.43 to 1.65)	0.89 (−0.34 to 2.13)
Non-Hispanic White	2702 (47.6)	4.9	1 [Reference]	1.64 (1.17 to 2.11)
**Age 30-39 y**				
Hispanic	9336 (19.8)	34.0	0.76 (0.74 to 0.78)	1.06 (0.55 to 1.58)
Non-Hispanic American Indian or Alaska Native	357 (0.8)	38.3	0.86 (0.77 to 0.95)	1.13 (−0.39 to 2.68)
Non-Hispanic Asian or Pacific Islander	5663 (12.0)	41.8	0.94 (0.91 to 0.96)	0.46 (−0.11 to 1.04)
Non-Hispanic Black	6732 (14.3)	51.3	1.15 (1.12 to 1.18)	0.54 (0.14 to 0.93)
Non-Hispanic White	25 099 (53.2)	44.7	1 [Reference]	0.56 (0.34 to 0.77)
**Age 40-49 y**				
Hispanic	26 414 (16.0)	112.6	0.75 (0.74 to 0.76)	0.72 (0.24 to 1.21)
Non-Hispanic American Indian or Alaska Native	1080 (0.7)	107.7	0.72 (0.68 to 0.76)	1.86 (0.60 to 3.14)
Non-Hispanic Asian or Pacific Islander	18 969 (11.5)	145.1	0.97 (0.96 to 0.99)	1.75 (1.11 to 2.39)
Non-Hispanic Black	19 325 (11.7)	143.5	0.96 (0.94 to 0.97)	0.20 (−0.06 to 0.45)
Non-Hispanic White	99 159 (60.1)	149.6	1 [Reference]	0.90 (0.55 to 1.26)

^a^ Average annual percent change was calculated in weighted sum of annual percent change sections.

^b^ Estimated annual percent changes could not be calculated due to small sample sizes.

### Differences in Breast Cancer Incidence Rates by Age Group, Race, and Hormone Receptor Status

Increases in ER+/PR+ and ER+/PR− tumors were observed across almost all age and racial groups (Table 3). Among women aged 20 to 29, non-Hispanic Black women had the highest ASIRs for all molecular subtypes, except for ER−/PR+ tumors, which was highest among non-Hispanic American Indian or Alaska Native, although it must be noted that this was based on very small numbers. Between ages 30 to 39 and 40 to 49 years, non-Hispanic Black women had the highest ASIRs for all molecular subtypes except for ER+/PR+, where ASIR was lower than for non-Hispanic White women (age 30-39 years: IRR, 0.90; 95% CI, 0.86 to 0.94; age 40-99 years: IRR, 0.69; 95% CI, 0.68 to 0.71).

**Table 3.** Trends in Breast Cancer Incidence Among Women Aged 20-49 Years by Race and Ethnicity, Hormone Receptor Status, and Age, 2000-2019

Characteristics	Total, No. (%)	Age-adjusted incidence rates, No. per 100 000	Incidence rate ratios (95% CI)	Average annual change, % (95% CI)^a^
**Age 20-29 y**				
ER+/PR+^b^				
Hispanic	534 (20.4)	1.7	0.73 (0.66 to 0.81)	5.06 (3.26 to 6.89)
Non-Hispanic American Indian or Alaska Native	18 (0.7)	1.7	0.71 (0.42 to 1.12)	NA^b^
Non-Hispanic Asian or Pacific Islander	312 (11.9)	2.4	0.99 (0.87 to 1.12)	4.17 (2.08 to 6.32)
Non-Hispanic Black	425 (16.3)	3.0	1.26 (1.13 to 1.41)	4.67 (3.22 to 6.15)
Non-Hispanic White	1325 (50.7)	2.4	1 [Reference]	4.02 (2.57 to 5.48)
ER+/PR−				
Hispanic	149 (24.6)	0.5	1.00 (0.81 to 1.22)	5.16 (2.45 to 7.94)
Non-Hispanic American Indian or Alaska Native	5 (0.8)	0.5	0.97 (0.31 to 2.28)	NA
Non-Hispanic Asian or Pacific Islander	50 (8.2)	0.4	0.77 (0.56 to 1.05)	NA
Non-Hispanic Black	132 (21.8)	0.9	1.91 (1.54 to 2.36)	2.71 (−0.90 to 6.44)
Non-Hispanic White	271 (44.7)	0.5	1 [Reference]	4.11 (1.24 to 7.06)
ER−/PR+				
Hispanic	25 (19.5)	0.1	0.72 (0.43 to 1.16)	NA
Non-Hispanic American Indian or Alaska Native	2 (1.6)	0.2	1.66 (0.20 to 6.21)	NA
Non-Hispanic Asian or Pacific Islander	14 (10.9)	0.1	0.93 (0.48 to 1.70)	NA
Non-Hispanic Black	24 (18.8)	0.2	1.50 (0.89 to 2.43)	NA
Non-Hispanic White	63 (49.2)	0.1	1 [Reference]	NA
ER−/PR−				
Hispanic	397 (23.3)	1.3	0.91 (0.80 to 1.02)	−0.16 (−1.28 to 0.98)
Non-Hispanic American Indian or Alaska Native	18 (1.1)	1.7	1.19 (0.70 to 1.88)	NA
Non-Hispanic Asian or Pacific Islander	149 (8.7)	1.1	0.78 (0.65 to 0.93)	−0.22 (−2.69 to 2.31)
Non-Hispanic Black	348 (20.4)	2.5	1.73 (1.52 to 1.96)	−0.46 (−2.49 to 1.62)
Non-Hispanic White	793 (46.5)	1.4	1 [Reference]	0.18 (−0.85 to 1.23)
**Age 30-39 y**				
ER+/PR+				
Hispanic	4627 (18.6)	17.0	0.69 (0.67 to 0.71)	3.39 (2.71 to 4.07)
Non-Hispanic American Indian or Alaska Native	186 (0.7)	20.0	0.81 (0.70 to 0.94)	3.24 (1.26 to 5.25)
Non-Hispanic Asian or Pacific Islander	3467 (13.9)	25.7	1.05 (1.01 to 1.09)	2.31 (1.64 to 2.98)
Non-Hispanic Black	2891 (11.6)	22.1	0.90 (0.86 to 0.94)	3.99 (3.12 to 4.86)
Non-Hispanic White	13 772 (55.2)	24.6	1 [Reference]	2.52 (1.91 to 3.13)
ER+/PR−				
Hispanic	921 (20.4)	3.3	0.81 (0.75 to 0.88)	2.79 (1.33 to 4.26)
Non-Hispanic American Indian or Alaska Native	31 (0.7)	3.3	0.81 (0.55 to 1.15)	NA
Non-Hispanic Asian or Pacific Islander	484 (10.7)	3.6	0.86 (0.78 to 0.95)	2.52 (0.57 to 4.51)
Non-Hispanic Black	750 (16.6)	5.7	1.38 (1.27 to 1.50)	4.17 (2.83 to 5.53)
Non-Hispanic White	2321 (51.5)	4.1	1 [Reference]	2.63 (2.06 to 3.22)
ER−/PR+				
Hispanic	179 (18.9)	0.6	0.71 (0.60 to 0.84)	1.70 (−1.39 to 4.88)
Non-Hispanic American Indian or Alaska Native	10 (1.1)	1.1	1.19 (0.57 to 2.20)	NA
Non-Hispanic Asian or Pacific Islander	97 (10.2)	0.7	0.78 (0.62 to 0.97)	−4.16 (−7.17 to −1.05)
Non-Hispanic Black	149 (15.7)	1.1	1.25 (1.03 to 1.50)	−2.62 (−5.88 to 0.76)
Non-Hispanic White	513 (54.1)	0.9	1 [Reference]	−3.08 (−4.54 to −1.59)
ER−/PR−				
Hispanic	2743 (21.2)	9.9	0.85 (0.81 to 0.89)	0.46 (−0.44 to 1.37)
Non-Hispanic American Indian or Alaska Native	103 (0.8)	11.0	0.94 (0.76 to 1.14)	NA
Non-Hispanic Asian or Pacific Islander	1192 (9.2)	8.8	0.75 (0.70 to 0.80)	−0.93 (−2.09 to 0.24)
Non-Hispanic Black	2283 (17.6)	17.4	1.48 (1.41 to 1.55)	−1.20 (−2.12 to −0.27)
Non-Hispanic White	6617 (51.1)	11.7	1 [Reference]	−0.85 (−1.42 to −0.27)
**Age 40-49 y**				
ER+/PR+				
Hispanic	16 312 (15.3)	69.5	0.69 (0.68 to 0.70)	2.97 (2.54 to 3.40)
Non-Hispanic American Indian or Alaska Native	685 (0.6)	68.2	0.68 (0.63 to 0.73)	3.60 (2.02 to 5.21)
Non-Hispanic Asian or Pacific Islander	13 243 (12.4)	101.3	1.01 (0.99 to 1.03)	3.16 (2.79 to 3.53)
Non-Hispanic Black	9368 (8.8)	69.6	0.69 (0.68 to 0.71)	3.27 (2.45 to 4.10)
Non-Hispanic White	66 859 (62.8)	100.6	1 [Reference]	2.69 (2.23 to 3.15)
ER+/PR−				
Hispanic	2054 (15.4)	8.8	0.86 (0.82 to 0.90)	2.05 (1.13 to 2.97)
Non-Hispanic American Indian or Alaska Native	78 (0.7)	7.8	0.76 (0.60 to 0.96)	1.55 (−1.58 to 4.78)
Non-Hispanic Asian or Pacific Islander	1318 (10.0)	10.1	0.99 (0.93 to 1.05)	1.36 (0.19 to 2.54)
Non-Hispanic Black	1824 (15.2)	13.5	1.33 (1.26 to 1.40)	3.06 (0.89 to 5.28)
Non-Hispanic White	6735 (58.7)	10.2	1 [Reference]	−0.11 (−0.75 to 0.54)
ER−/PR+				
Hispanic	399 (16.4)	1.7	0.81 (0.72 to 0.91)	−5.26 (−7.75 to −2.71)
Non-Hispanic American Indian or Alaska Native	16 (0.7)	1.6	0.77 (0.44 to 1.26)	NA
Non-Hispanic Asian or Pacific Islander	227 (9.3)	1.7	0.83 (0.72 to 0.95)	−3.56 (−6.00 to −1.06)
Non-Hispanic Black	410 (16.9)	3.1	1.46 (1.30 to 1.63)	−1.84 (−3.96 to 0.32)
Non-Hispanic White	1378 (56.7)	2.1	1 [Reference]	−3.66 (−5.14 to −2.16)
ER−/PR−				
Hispanic	5255 (17.3)	22.4	0.90 (0.87 to 0.93)	−0.63 (−1.28 to 0.02)
Non-Hispanic American Indian or Alaska Native	220 (0.7)	22.0	0.89 (0.77 to 1.01)	−0.70 (−2.93 to 1.58)
Non-Hispanic Asian or Pacific Islander	2786 (9.2)	21.3	0.86 (0.82 to 0.89)	−0.81 (−1.57 to −0.04)
Non-Hispanic Black	5810 (19.1)	43.1	1.73 (1.68 to 1.79)	−0.12 (−1.46 to 1.23)
Non-Hispanic White	16 371 (53.8)	24.9	1 [Reference]	−1.33 (−1.73 to −0.93)

Abbreviations: ER, estrogen receptor; PR, progesterone receptor; NA, not available.

^a^ Average annual percent change was calculated in weighted sum of annual percent change sections.

^b^ Estimated annual percent changes cannot be calculated due to small sample sizes.

### Cohort and Period Effects on Breast Cancer Incidence

**Figure 2.**
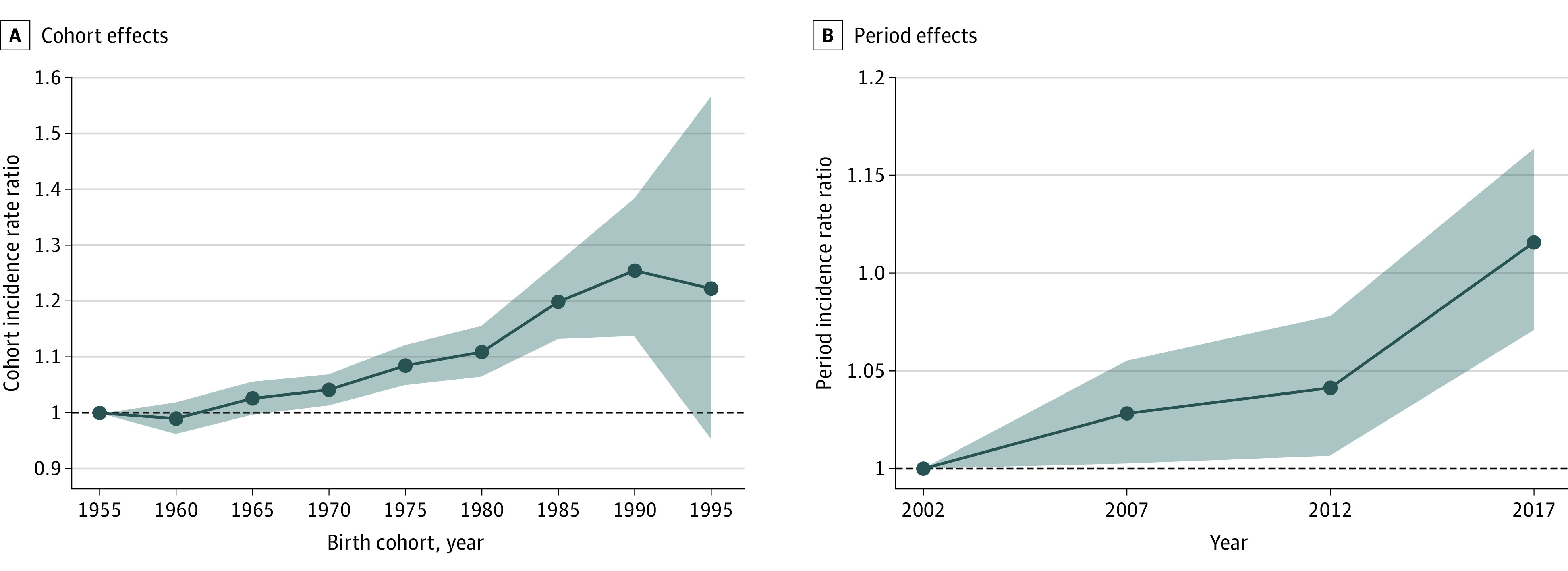
Cohort and Period Effects on Overall Incidence of Primary Invasive Breast Cancer Among US Women Aged 20 to 49 Years Shaded areas indicate 95% CIs. In panel B, midyear point denotes the 5-year period: 2002 year denotes period 2000 to 2004; 2007 year denotes period 2005 to 2009; 2012 year denotes period 2010 to 2014; and 2017 year denotes period 2015 to 2019.

The age-period-cohort models indicated strong period (*P* = 4.77 × 10^−9^) and cohort (*P* = 4.83 × 10^−14^) effects on breast cancer incidence. The cohort effect was approximately 2 times larger than the period effect. Incidence rates by birth cohort increased across the study period with peaks in the 1990 (IRR, 1.26; 95% CI, 1.14 to 1.38) and 1995 (IRR, 1.22; 95% CI, 0.95 to 1.57) birth cohorts (Figure 2). Incidence rates by calendar periods rose and peaked in the period from 2015 to 2019 (IRR, 1.12; 95% CI, 1.07 to 1.16). Incidence rate by birth cohort peaked for the 1990 birth cohort for non-Hispanic White (IRR, 1.34; 95% CI, 1.19 to 1.51) and non-Hispanic American Indian or Alaska Native women (IRR, 1.56; 95% CI, 0.79 to 3.08). Incidence rates by both period and cohort increased for ER+/PR+ and ER+/PR− tumors but decreased for ER−/PR+ and ER−/PR− tumors. Recent periods (2015-2019) and birth cohorts (1995) had lower incidence rates for stage II (period IRR, 0.97; 95% CI, 0.93 to 1.01; cohort IRR, 0.97; 95% CI, 0.76 to 1.25) and III (period IRR, 0.82; 95% CI, 0.77 to 0.88; cohort IRR, 0.49; 95% CI, 0.31 to 0.77) tumors, but higher incidence rates for stage I (period IRR, 1.39; 95% CI, 1.25 to 1.55; cohort IRR, 1.70; 95% CI, 0.89 to 3.26) and IV tumors (period IRR, 1.93; 95% CI, 1.75 to 2.12; cohort IRR, 5.60; 95% CI, 3.44 to 9.11) (eFigures 4,5, and 6 in Supplement 1).

## Discussion

We described trends in breast cancer incidence rates among women aged 20 to 49 years over the past 20 years as well as the impact of calendar period and birth cohort effects on these incidence rates by race and ethnicity, HR, and stage. We observed an increase in incidence rates among women aged 20 to 49 years, especially after 2016. Non-Hispanic Black women aged between 20 to 29 and 30 to 39 years had higher incidence rates than all other race and ethnicity groups. Incidence rates for ER+ tumors increased while those for ER− tumors decreased. Incidence rates for stage II and III tumors decreased, while those for stages I and IV tumors increased.

Our findings of increased incidence rates among young US women are consistent with previous studies.^13,14,15^ Non-Hispanic Black women aged 20 to 29 and 30 to 39 years had the highest ASIR with a rising trend of ASIR and cohort and period effects. However, the incidence rate was highest among non-Hispanic White women aged 40 to 49 years. Similar to our findings, a study using SEER data that looked at trends over a shorter time period (from 2004 to 2013) found that breast cancer incidence was highest among non-Hispanic Black women, especially for women aged under 45 years.^7^ The differences in age-related breast cancer incidence rates between non-Hispanic Black and non-Hispanic White women, with a crossover occurring at age 40 years has been previously described^16^ and requires further studies to understand factors driving this phenomenon. Non-Hispanic Black women also had the highest incidence for advanced staged disease, which likely contributes to their higher mortality rates.^17,18,19^ Our findings suggest that breast cancer risk assessment should start at an early age in non-Hispanic Black women to determine whether targeted screening should be recommended earlier in non-Hispanic Black women at high-risk.

Hispanic women have younger ages at first birth, higher maternal parity, and breastfeed for longer periods when compared with non-Hispanic White women,^20,21^ which may contribute to them having one of the lowest incidence rates we observed in our study. However, this may be changing as fertility rates are falling and age at first birth is rising among next-generation Hispanic women which affects their risk.^22,23,24^ The increase in incidence rates among Hispanic women was greater than among women of other races and ethnicities with substantial increases in both cohort and period risks. A migration study found that increased risks of breast cancer among Hispanic women were associated with longer residence time in the US and increasing acculturation.^25^

Our results by hormone receptor status are consistent with previous studies on incidence trends.^8,26,27^ Trends in reproductive risk factors could explain the increasing trend in ER+ tumors and the decreasing trends in ER− tumors. The age at first birth for US women has increased from 1970 to 2017 across all races and ethnicities.^28^ Older age at first birth has been associated with increased risk of ER+/PR+ tumors and reduced risk of ER−/PR− tumors.^29,30^ Decreased parity number is another reproductive factor associated with increased risks of ER+ tumor among young women^29,31^ and the number of live births has decreased in the US from 1966 to 2015.^31^ Obesity may also be one of the possible reasons for the decrease in ER− tumor among young US women. A pooled study found that adult body mass index was inversely associated with risk of ER−/PR− breast cancer among premenopausal women.^32^ An increasing trend in prevalence of adult obesity or overweight has been recorded across the study period.^33,34^

We observed the increasing incidence of stage I tumors, similar to what has been reported previously.^18^ This increase may be due to young women detecting tumors sooner than they would have in previous decades. The introduction of public health campaigns to provide young women with information about breast cancer risks and signs as well as the introduction of genetic testing for BRCA mutations and subsequent close surveillance of positive patients may be responsible for this earlier detection.^35^ We also found an increase in the incidence of stage IV tumors, consistent with that of a previous study.^36^ The increase was also observed across birth cohorts. Screening for women who are not at an elevated risk breast cancer is not recommended before age 40 years,^37^ so young women often detect their own breast cancers, which may lead to them not being discovered until they have reached a more advanced stage.^38^ Obesity is another risk factor for advanced stage breast cancer which has evolved over successive birth cohorts as the prevalence of obesity has significantly increased in the US.^39^ Although a higher body mass index is associated with reduced risk of premenopausal breast cancer, a previous study reported a positive association between body mass and advanced stage at diagnosis, especially in young women.^40^

In age-period cohort analysis, we observed that the increases in incidence rates were mostly explained by cohort effects although there were also significant period effects for most races and ethnicities. The presence of period and cohort effects across races and ethnicities suggests that these disparities cannot simply be accounted for by intrinsic biological differences alone. It is important to evaluate the roles of social determinants of health that affect breast cancer risk as these could have implications for prevention by identifying modifiable risk factors. Lower maternal parity,^31^ older age at first birth,^28,41^ more individuals with family history of breast cancer,^42^ earlier age at menarche^43^ as well as increased alcohol intake^44^ may contribute to the increase in breast cancer incidence.^3,45,46,47^

### Strengths and Limitations

To the best of our knowledge, our study is one of the first study to explore the calendar period and birth cohort effects on breast cancer incidence among US women aged 20 to 49 years through comprehensive analysis of the SEER 17 databases in conjunction with examining the most recent 20-year breast cancer incidence trends by race and ethnicity, HR, and stage.

This study had several limitations. First, we excluded missing data for unknown ER or PR status, which could lead to underestimated ASIRs. Second, ERBB2 information became available in SEER data set from 2010, hence we could not evaluate more intricate molecular subtypes over the 20-year period. Third, SEER does not provide information of risk factor data.

## Conclusions

In this population-based cross-sectional study, breast cancer incidence rates in the US increased among women aged 20 to 49 years, driven mainly by increases in the incidence of ER+ tumors, as incidence of ER− tumors decreased. Also notable are the elevated incidence rates among non-Hispanic Black women aged 20 to 29 and 30 to 39 years. Our findings underscore the need for further research into specific breast cancer risk factors among younger women and possible targeted breast cancer prevention strategies for at-risk groups.

## Notes

Supplement 1. **eFigure 1.** Joinpoint Analysis of Primary Invasive Breast Cancer Incidence by Race and Ethnicity Among US Women Aged 20 to 49 Years Between 200 and 2019, SEER 17 **eFigure 2.** Joinpoint Analysis of Primary Invasive Breast Cancer Incidence by Hormone Receptor Status Among US Women Aged 20 to 49 Years Between 200 and 2019, SEER 17 **eFigure 3.** Joinpoint Analysis of Primary Invasive Breast Cancer Incidence by Tumor Stage Among US Women Aged 20 to 49 Years Between 200 and 2019, SEER 17 **eFigure 4.** Cohort and Period Effects on Primary Invasive Breast Cancer Incidence by Race and Ethnicity Among US Women Aged 20 to 49 Years Between 200 and 2019, SEER 17 **eFigure 5.** Cohort and Period Effects on Primary Invasive Breast Cancer Incidence by Hormone Receptor Status Among US Women Aged 20 to 49 Years Between 200 and 2019, SEER 17 **eFigure 6.** Cohort and Period Effects on Primary Invasive Breast Cancer Incidence by Tumor Stage Among US Women Aged 20 to 49 Years Between 200 and 2019, SEER 17

Supplement 2.
Data Sharing Statement
