# Supplementary material for: Breast Cancer Incidence Among US Women Aged 20 to 49 Years by Race, Stage, and Hormone Receptor Status
Source: JAMA Netw Open. 2024 Jan 26;7(1):e2353331. doi: 10.1001/jamanetworkopen.2023.53331 (PMC10818222; doi:10.1001/jamanetworkopen.2023.53331)
Supplement: Supplement 1. — eFigure 1. Joinpoint Analysis of Primary Invasive Breast Cancer Incidence by Race and Ethnicity Among US Women Aged 20 to 49 Years Between 200 and 2019, SEER 17 eFigure 2. Joinpoint Analysis of Primary Invasive Breast Cancer Incidence by Hormone Receptor Status Among US Women Aged 20 to 49 Years Between 200 and 2019, SEER 17 eFigure 3. Joinpoint Analysis of Primary Invasive Breast Cancer Incidence by Tumor Stage Among US Women Aged 20 to 49 Years Between 200 and 2019, SEER 17 eFigure 4. Cohort and Period Effects on Primary Invasive Breast Cancer Incidence by Race and Ethnicity Among US Women Aged 20 to 49 Years Between 200 and 2019, SEER 17 eFigure 5. Cohort and Period Effects on Primary Invasive Breast Cancer Incidence by Hormone Receptor Status Among US Women Aged 20 to 49 Years Between 200 and 2019, SEER 17 eFigure 6. Cohort and Period Effects on Primary Invasive Breast Cancer Incidence by Tumor Stage Among US Women Aged 20 to 49 Years Between 200 and 2019, SEER 17 [file jamanetwopen-e2353331-s001.pdf]

Supplemental Online Content

Xu S, Murtagh S, Han Y, Wan F, Toriola AT. Breast cancer incidence among young women aged 20-49 years in the United States by race, stage, and hormone receptor status. *JAMA Netw Open.* 2024;7(1):e2353331. doi:10.1001/jamanetworkopen.2023.53331

eFigure 1. Joinpoint Analysis of Primary Invasive Breast Cancer Incidence by Race and Ethnicity Among US Women Aged 20 to 49 Years Between 200 and 2019, SEER 17

eFigure 2. Joinpoint Analysis of Primary Invasive Breast Cancer Incidence by Hormone Receptor Status Among US Women Aged 20 to 49 Years Between 200 and 2019, SEER 17

eFigure 3. Joinpoint Analysis of Primary Invasive Breast Cancer Incidence by Tumor Stage Among US Women Aged 20 to 49 Years Between 200 and 2019, SEER 17

eFigure 4. Cohort and Period Effects on Primary Invasive Breast Cancer Incidence by Race and Ethnicity Among US Women Aged 20 to 49 Years Between 200 and 2019, SEER 17

eFigure 5. Cohort and Period Effects on Primary Invasive Breast Cancer Incidence by Hormone Receptor Status Among US Women Aged 20 to 49 Years Between 200 and 2019, SEER 17

eFigure 6. Cohort and Period Effects on Primary Invasive Breast Cancer Incidence by Tumor Stage Among US Women Aged 20 to 49 Years Between 200 and 2019, SEER 17

This supplemental material has been provided by the authors to give readers additional information about their work.

© 2023 Xu S et al. *JAMA Network Open.*

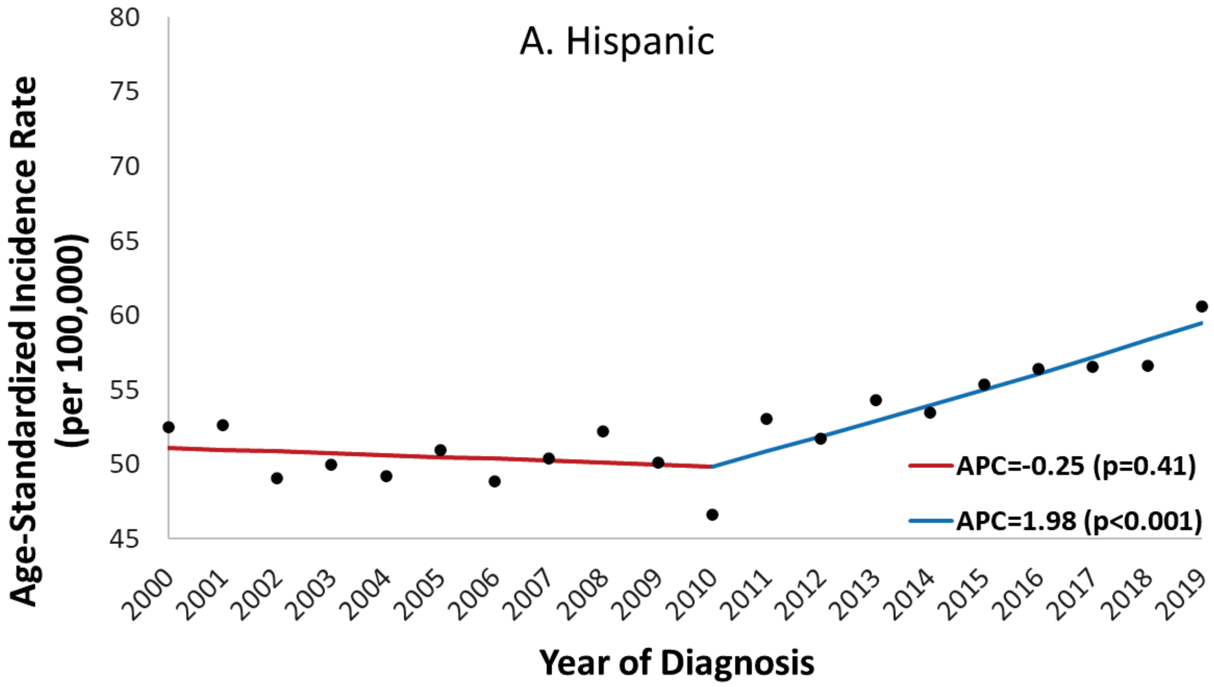

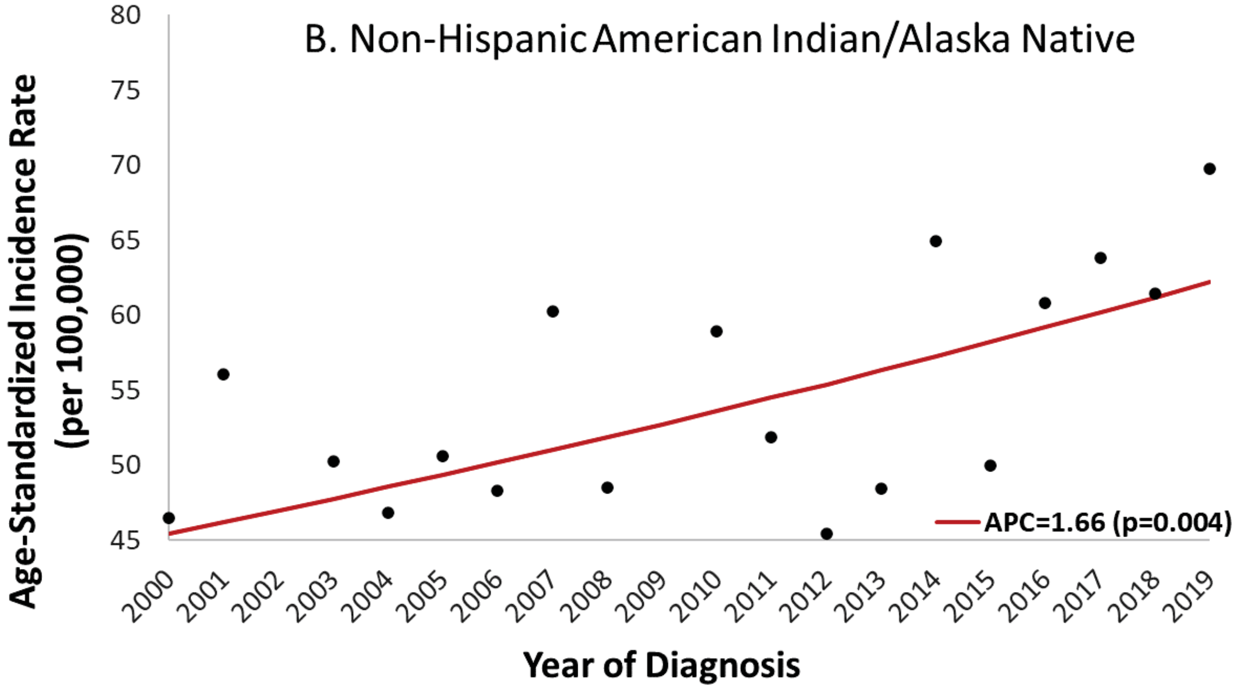

© 2023 Xu S et al. *JAMA Network Open*.

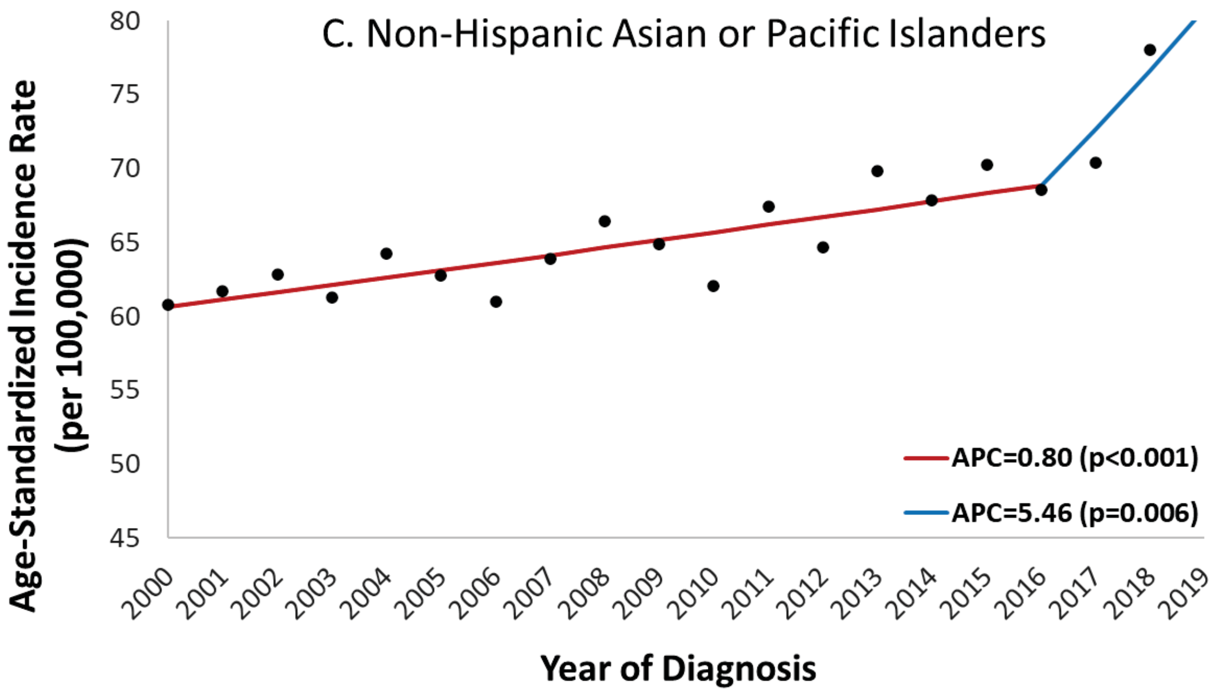

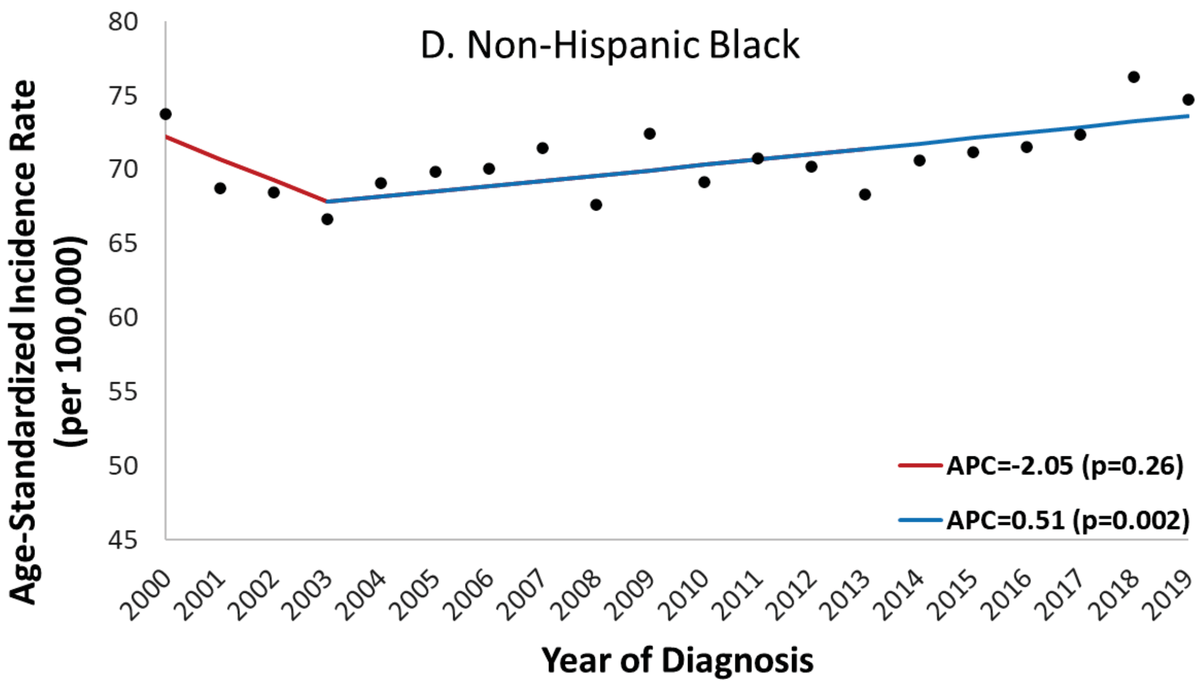

© 2023 Xu S et al. *JAMA Network Open*.

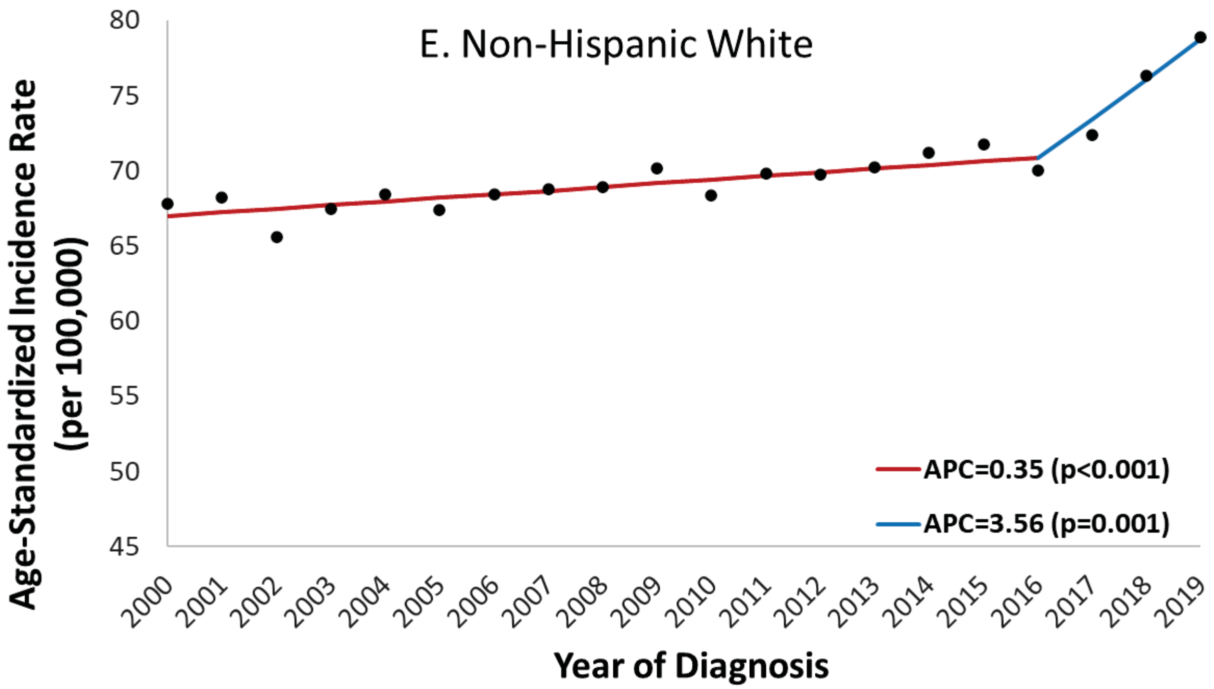

eFigure 1. Joinpoint Analysis of Primary Invasive Breast Cancer Incidence by Race/ Ethnicity (A. Hispanic; B. Non-Hispanic American Indian/Alaska Native; C. Non-Hispanic Asian or Pacific Islanders; D. Non-Hispanic Black; E. Non-Hispanic White) among US women aged 20 to 49 years between 2000 and 2019, SEER 17

APC = annual percent changes (%)

© 2023 Xu S et al. *JAMA Network Open*.

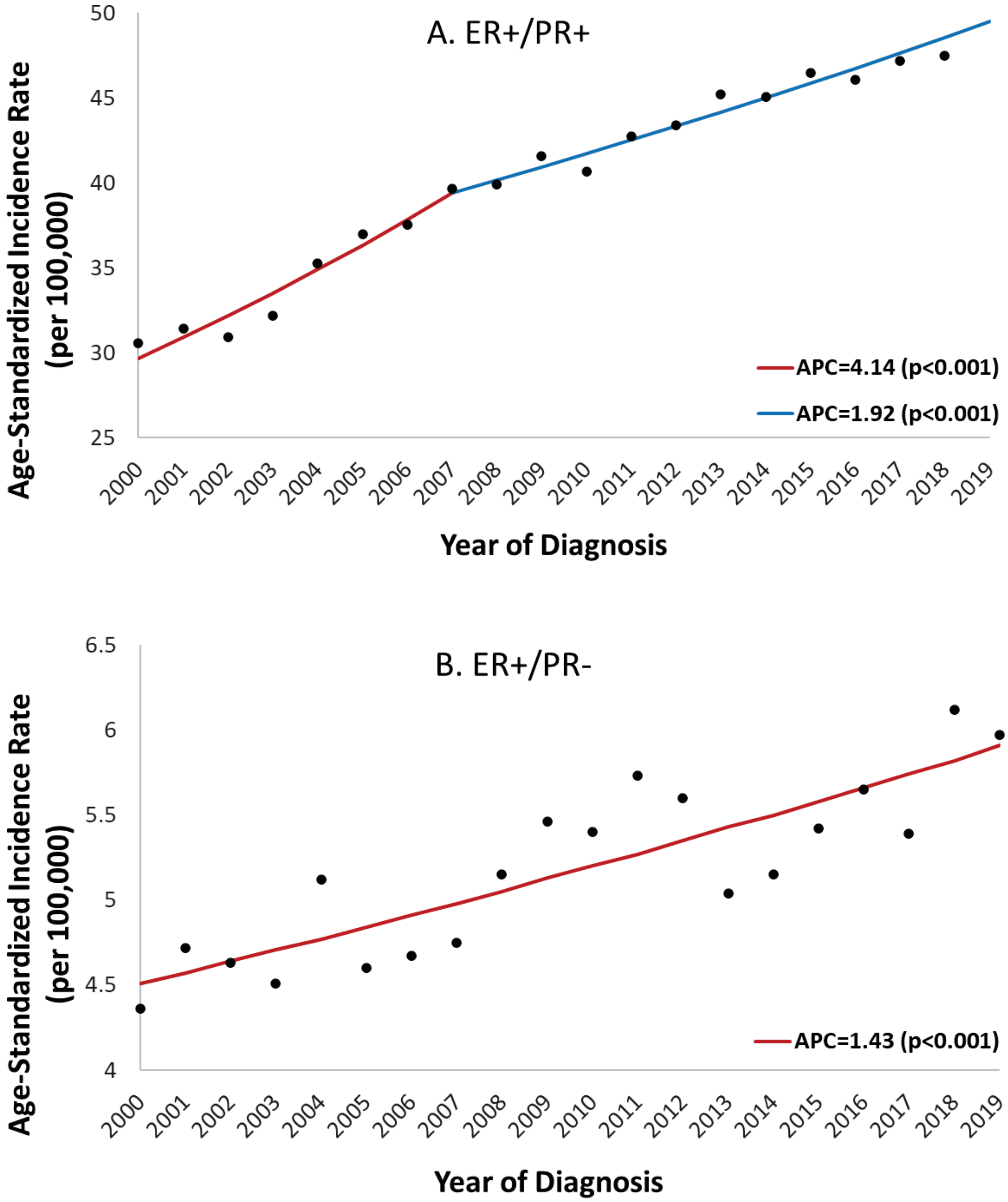

© 2023 Xu S et al. *JAMA Network Open*.

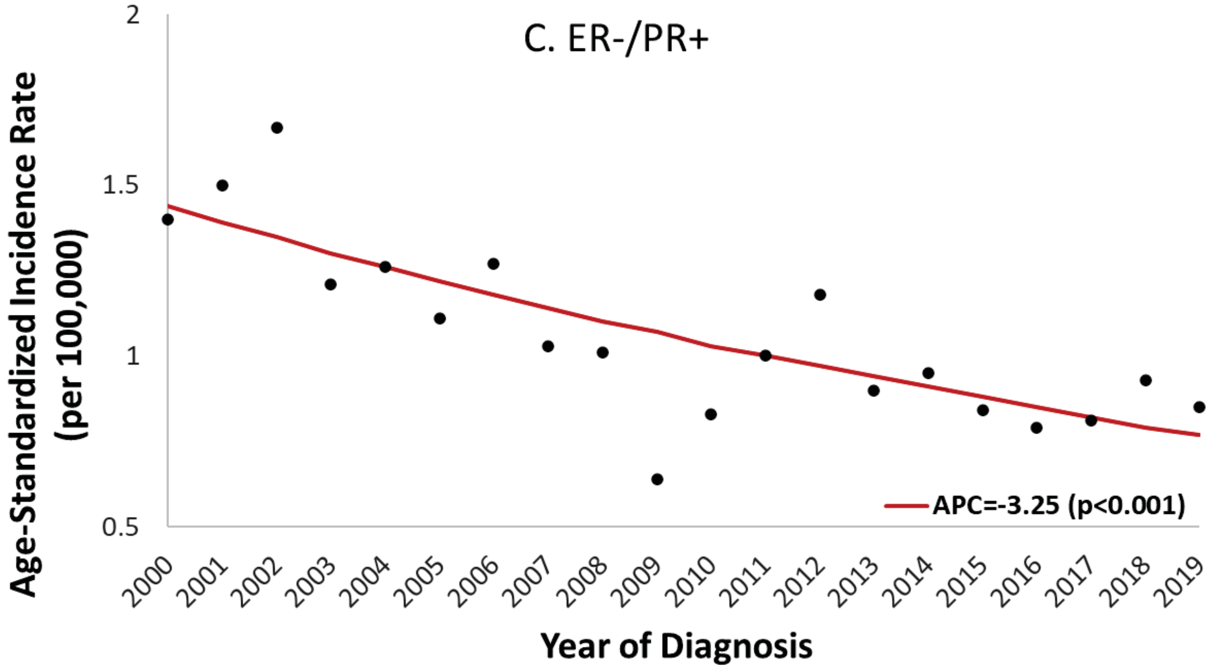

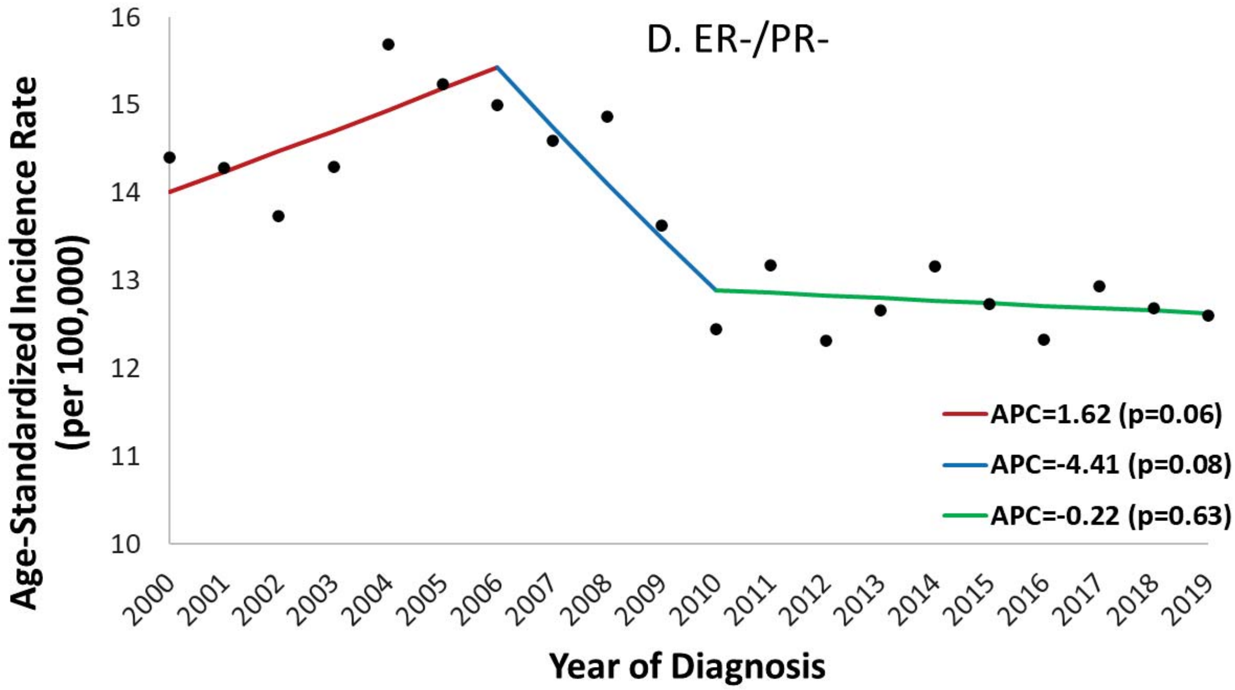

eFigure 2. Joinpoint Analysis of Primary Invasive Breast Cancer Incidence by Hormone Receptor Status (A. ER+/PR+; B. ER+/PR-; C. ER-/PR+; D. ER-/PR-) among US women aged 20 to 49 years between 2000 and 2019, SEER 17

APC = annual percent changes (%); ER = estrogen receptor; PR = progesterone receptor

© 2023 Xu S et al. *JAMA Network Open.*

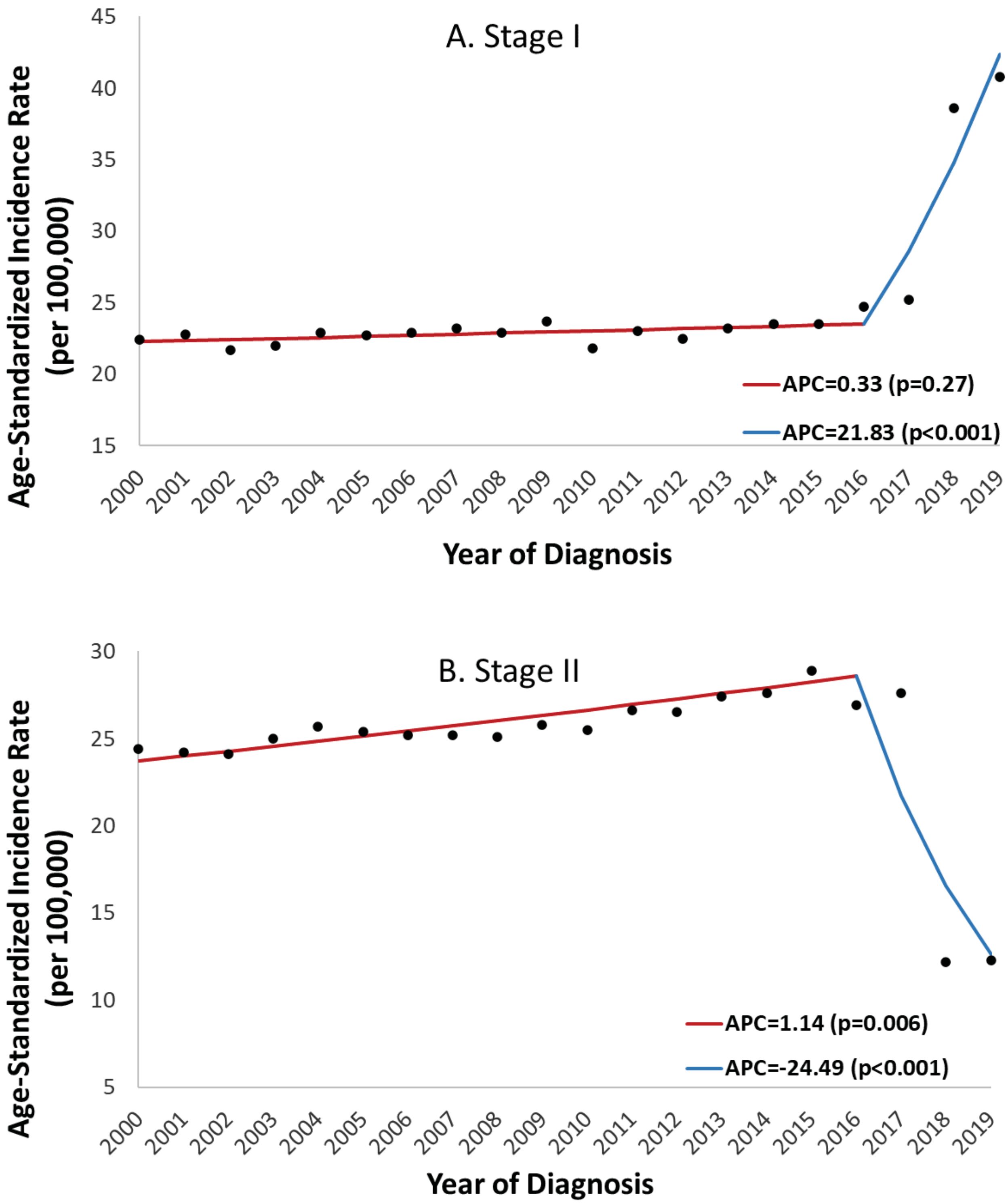

© 2023 Xu S et al. *JAMA Network Open*.

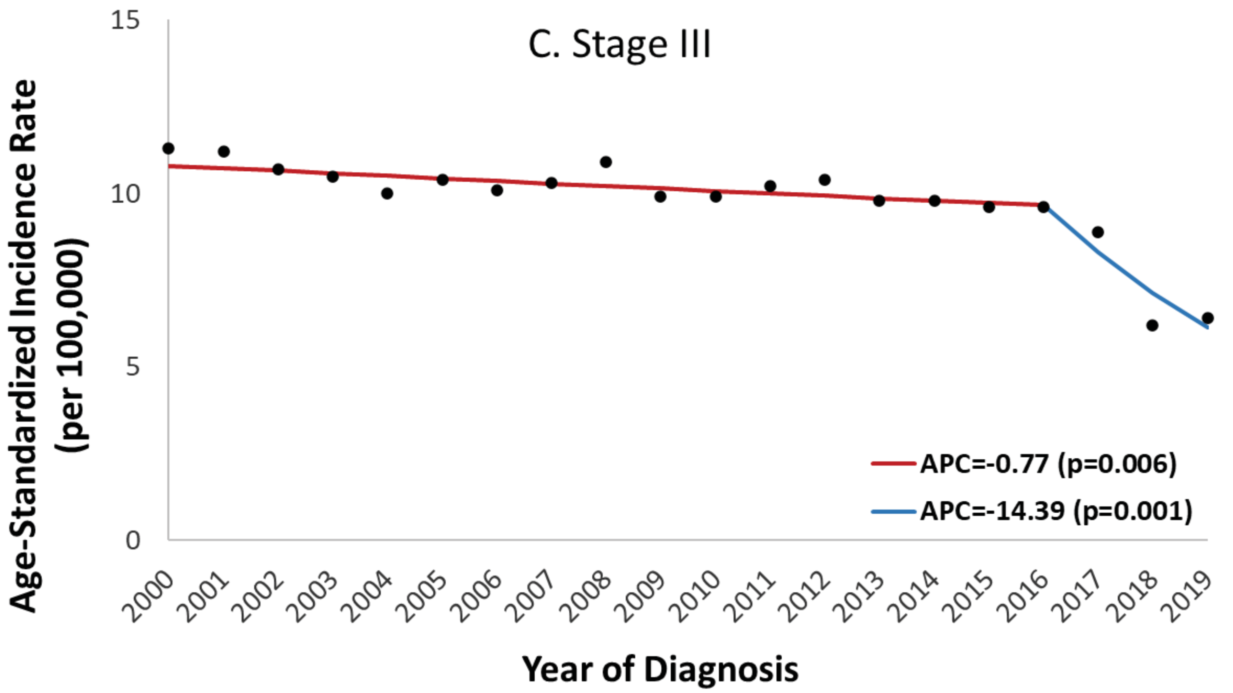

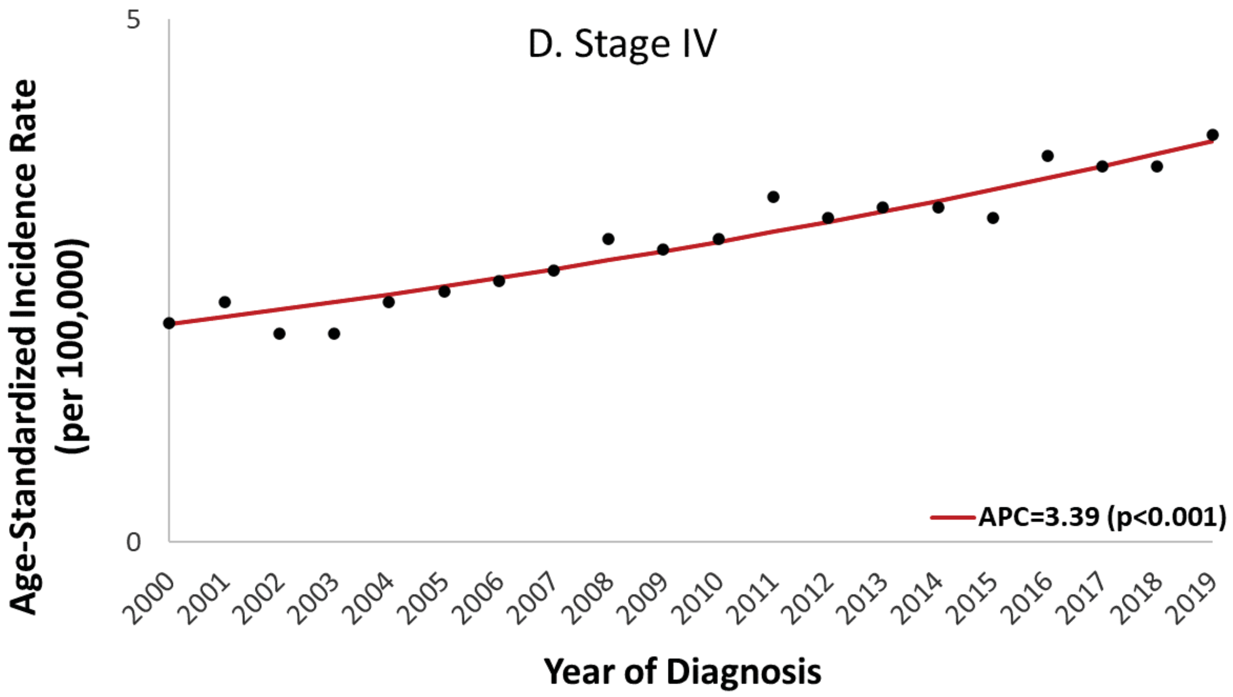

eFigure 3. Joinpoint Analysis of Primary Invasive Breast Cancer Incidence by Tumor Stage (A. stage I; B. stage II; C. stage III; D. stage IV) among US women aged 20 to 49 years between 2000 and 2019, SEER 17

APC = annual percent changes (%); ER = estrogen receptor; PR = progesterone receptor

© 2023 Xu S et al. *JAMA Network Open*.

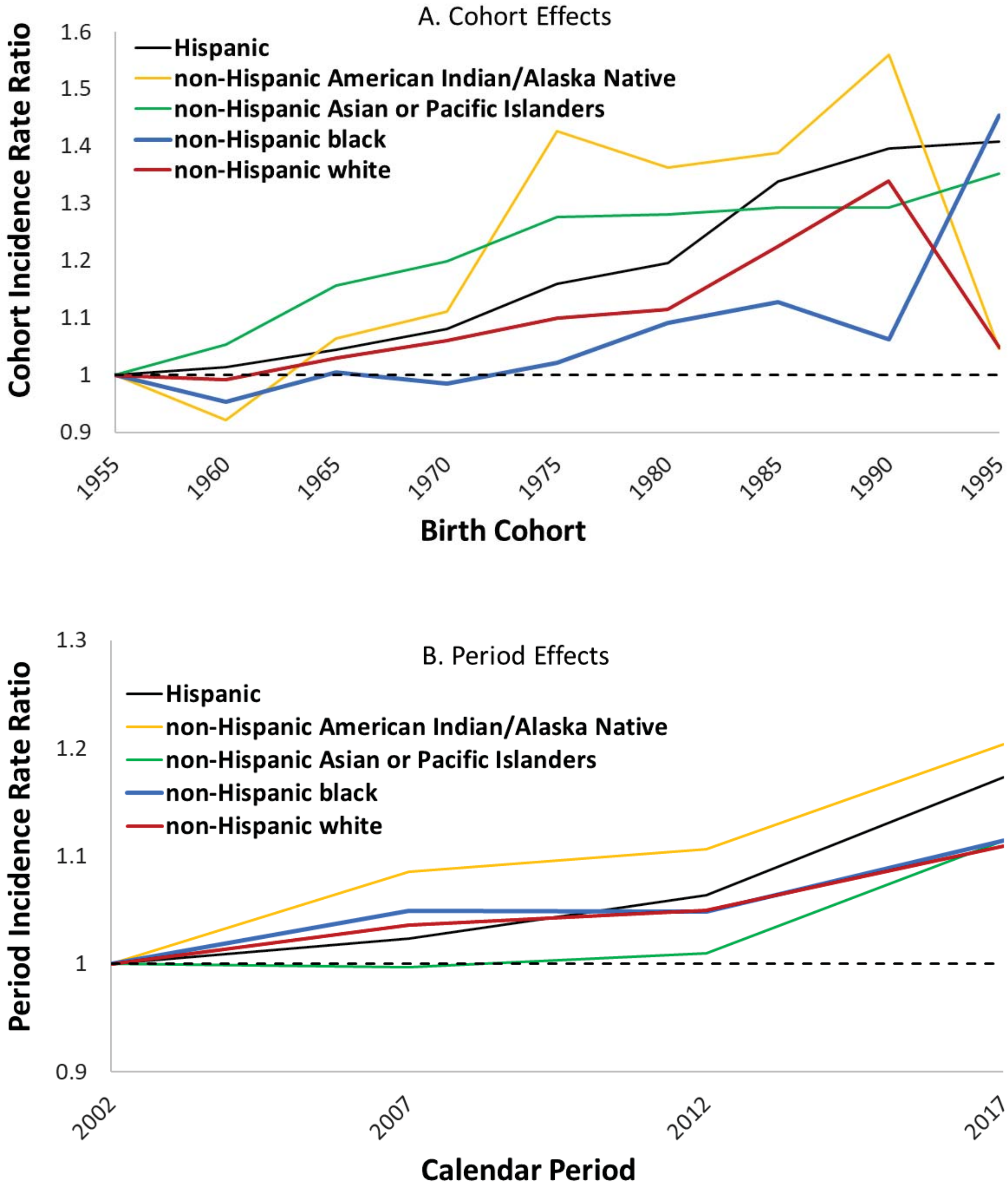

eFigure 4. Cohort (A) and Period (B) effects on Primary Invasive Breast Cancer Incidence by Race/ Ethnicity among US women aged 20 to 49 years, SEER 17

© 2023 Xu S et al. *JAMA Network Open*.

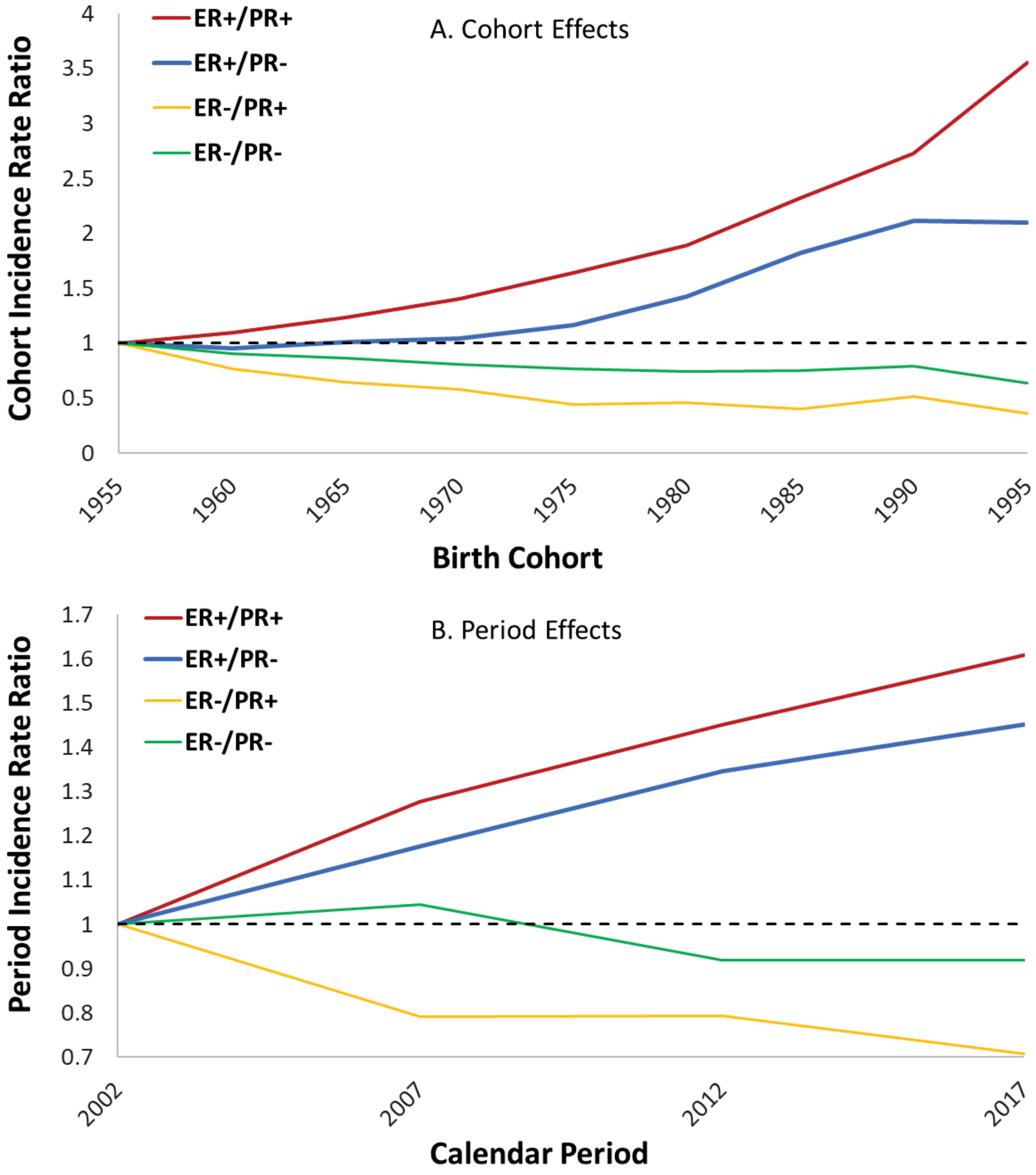

eFigure 5. Cohort (A) and Period (B) effects on Primary Invasive Breast Cancer Incidence by Hormone Receptor Status among US women aged 20 to 49 years, SEER 17

ER = estrogen receptor; PR = progesterone receptor

© 2023 Xu S et al. *JAMA Network Open*.

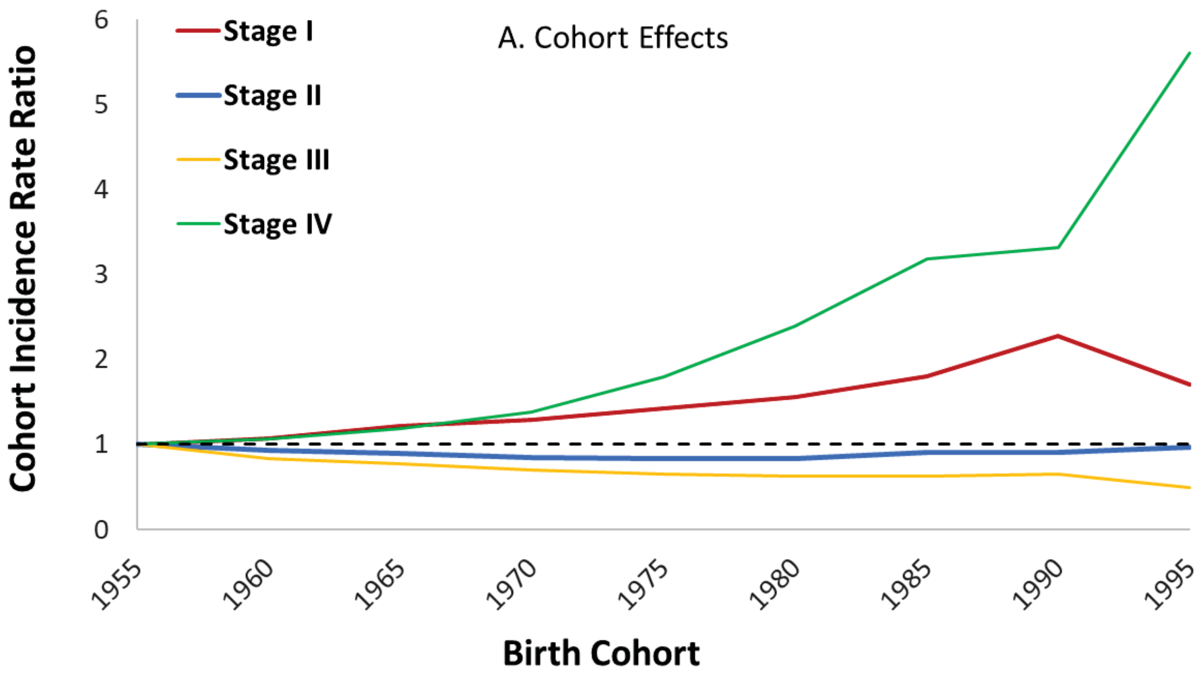

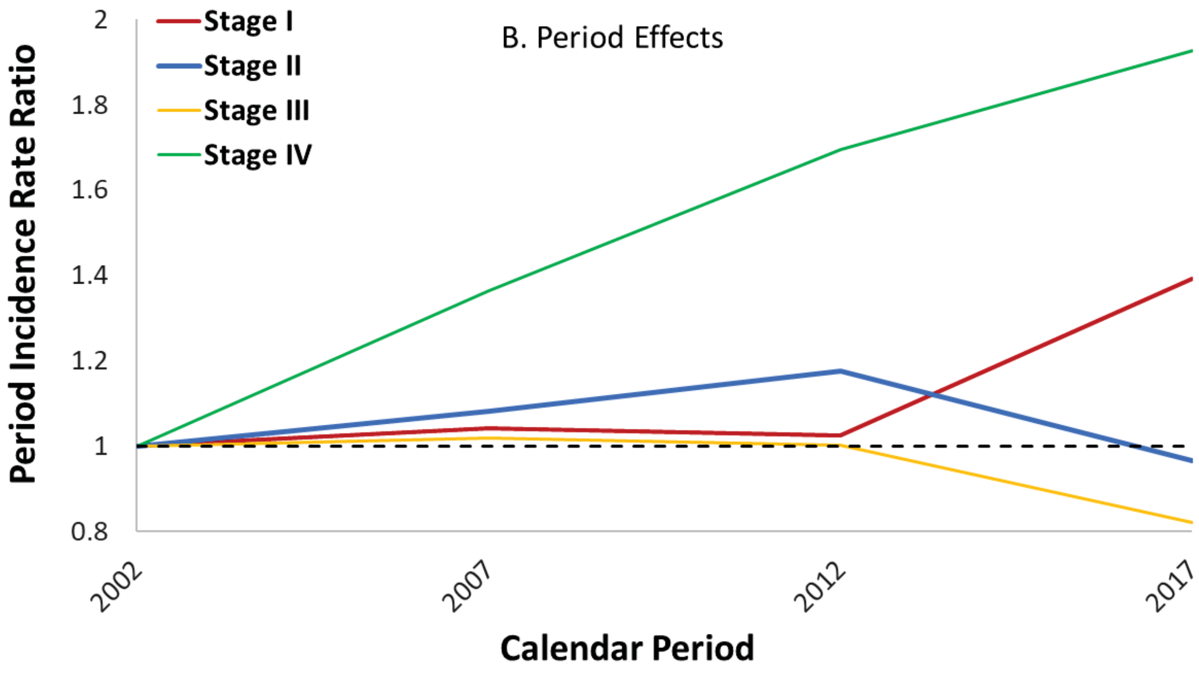

eFigure 6. Cohort (A) and Period (B) effects on Primary Invasive Breast Cancer Incidence by Tumor Stage among US women aged 20 to 49 years, SEER 17

© 2023 Xu S et al. *JAMA Network Open*.
