# Supplementary material for: Breast Cancer Incidence Among US Women Aged 20 to 49 Years by Race, Stage, and Hormone Receptor Status
Source: JAMA Netw Open. 2024 Jan 26;7(1):e2353331. doi: 10.1001/jamanetworkopen.2023.53331 (PMC10818222; doi:10.1001/jamanetworkopen.2023.53331)
Supplement: Supplement 2. — Data Sharing Statement [file jamanetwopen-e2353331-s002.pdf]

Data Sharing Statement

Xu. Breast Cancer Incidence Among US Women Aged 20 to 49 Years by Race, Stage, and Hormone Receptor Status. *JAMA Netw Open*. Published January 26, 2024.
doi:10.1001/jamanetworkopen.2023.53331

Data
Data available: No

Additional Information
Explanation for why data not available: Surveillance, Epidemiology, and End Results (SEER) data is publicly available. Requests for SEER data are to be made at https://seer.cancer.gov/data/access.html
